# CO_**2**_ Mitigation Measures of Power Sector and Its Integrated Optimization in China

**DOI:** 10.1100/2012/907685

**Published:** 2012-11-06

**Authors:** Pan Dai, Guang Chen, Hao Zhou, Meirong Su, Haixia Bao

**Affiliations:** ^1^College of Electrical Engineering, Zhejiang University, Hangzhou 310027, China; ^2^State Key Laboratory of Water Environment Simulation, School of Environment, Beijing Normal University, Beijing 100875, China

## Abstract

Power sector is responsible for about 40% of the total CO_2_ emissions in the world and plays a leading role in climate change mitigation. In this study, measures that lower CO_2_ emissions from the supply side, demand side, and power grid are discussed, based on which, an integrated optimization model of CO_2_ mitigation (IOCM) is proposed. Virtual energy, referring to energy saving capacity in both demand side and the power grid, together with conventional energy in supply side, is unified planning for IOCM. Consequently, the optimal plan of energy distribution, considering both economic benefits and mitigation benefits, is figured out through the application of IOCM. The results indicate that development of demand side management (DSM) and smart grid can make great contributions to CO_2_ mitigation of power sector in China by reducing the CO_2_ emissions by 10.02% and 12.59%, respectively, in 2015, and in 2020.

## 1. Introduction

Global climate change is a salient challenge in achieving sustainable development of human society. CO_2_ emissions and other greenhouse gas are the leading cause of global warming. If we do not take measures, CO_2_ emissions related to fuel energy will be doubled in 2050. As per statistical results of International Energy Agency (IEA), power sector is responsible for about 40% of the total CO_2_ emissions [1]. Consequently, CO_2_ mitigation in power sector is of great significance in achieving global mitigation goal.

Power sector can be divided into supply side, demand side and power grid according to transmission process. The CO_2_ emissions of power sector are concentrated in supply side, where fossil fuels burn. Unreasonable utilization in demand side and losses in power grid would increase energy consumption in supply side, which also indirectly contributes more CO_2_ emissions. CO_2_ mitigation measures adopted in supply side could be divided into three categories [2–8]: (a) improving conversion efficiency of fossil energy and lower energy intensity; (b) developing nonfossil energy like renewable energy and nuclear energy and adjust energy mix; (c) developing carbon capture and storage (CCS) technologies. The most effective measure in CO_2_ mitigation in demand side is to implement DSM, which improves utilization efficiency through incentive policies [9, 10]. The literature [11, 12] shows that DSM could reduce energy consumption by 5% to 15%. Power grid is not only a bridge connecting supply side and demand side physically, but also an important medium of achieving mitigation benefits of both sides. Besides, it provides support for large-scale applications of nonfossil energy (including nuclear energy, hydroelectric energy, and wind energy) [12]. With the development of smart grid and ultra-high voltage grid (UHV), losses decreased vastly. Thereby, power grid shows greater mitigation potential compared with other energy transmission methods. Based on current energy mix and generation technology, low-carbon power dispatch is an effective way to control CO_2_ emissions in a short period of time [13, 14].

The current researches on CO_2_ mitigation measures mainly focus on application of various mitigation techniques and macroinfluence of policies. However, researches on optimization of CO_2_ mitigation from the point of whole power sector are still rare. Integrated resource planning (IRP) and integrated resource strategic planning (IRSP) minimize power supply through optimization on both demand and supply sides [15–18]. However, energy saving capacity of power grid is neglected. In this study, an optimization model IOCM, considering all mitigation potential of supply side, demand side, and the power grid, is proposed and applied to the power sector in China.

In Section 2, the status quo of CO_2_ emissions of power sector is briefly represented. In Section 3, various measures on CO_2_ mitigation are discussed. Then, the IOCM is proposed in Section 4, and the result of IOCM applied to power sector in China is analyzed in Section 5. Finally, conclusions are made in Section 6.

## 2. CO_2_ Emissions of Power Sector in China

The major CO_2_ emissions country in the world, China, officially pledged to reduce its CO_2_ intensity by 40–45% from the 2005 level and increase the share of nonfossil energy in primary energy to 15% by 2020 [19]. CO_2_ emissions from power sector reached 3294.7 million tonnes (Mt) in 2009, accounting for 48% in total emissions [1]. Consequently, CO_2_ mitigation in power sector is of great significance in achieving long-term mitigation goal in China and even making contribution to global mitigation.

**Figure 1:**
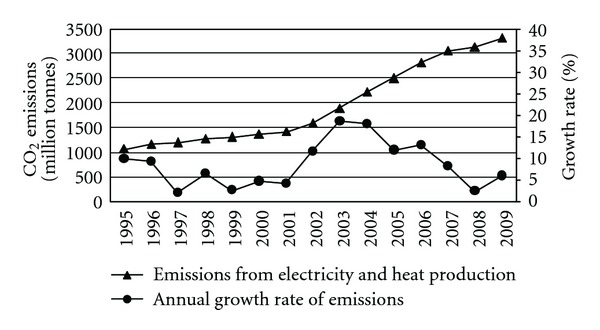
CO_2_ emissions from electricity and heat production from 1995 to 2009 in China.

China is at critical stage of industrialization and urbanization, and the demand for electricity increases rapidly. Electricity generation reached 4721.7 terawatt hours (TWh) in China in 2011, ranking in the second place in the world. Meanwhile, primary fuel mix is dominated by coal in China. Electricity from coal-fired power plants accounts for approximately 80% of the total electricity generation [20]. Therefore, the demand for electricity in China was the largest driver of the rise in emissions. As per statistical results of IEA, the CO_2_ emissions from electricity and heat production increased by 210% from 1,072.0 to 3,324.3 Mt between 1995 and 2009 [1], as shown in Figure 1.

**Figure 2:**
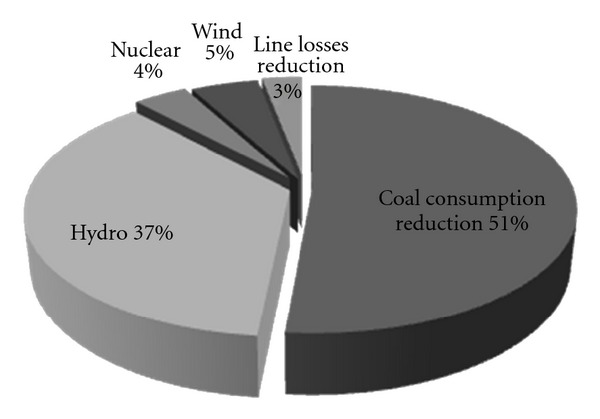
The contribution ratio of measures on CO_2_ mitigation from 2006 to 2010.

The primary responsibility of power sector is to ensure sufficient, safe, and stable power supply. Development is still the primary task, so effective measures should be taken to reduce CO_2_ emission, under the premise of meeting the power demand of economic and social development. Among the period of the “Eleventh Five-Year Plan” (from 2006 to 2010), measures to develop nonfossil energy, reduce coal consumption and line losses, and so forth, power sector has cut 1.74 billion tonnes of CO_2_. The contribution ratio of various measures for CO_2_ mitigation is displayed in Figure 2, among which measures to reduce coal consumption ranked at the top, up to 51% [21]. Although some achievements have been made concerning CO_2_ mitigation, a comparatively big gap from the target still exists. Therefore, various measures for CO_2_ mitigation should be promoted.

## 3. CO_2_ Mitigation Measures of Power Sector

### 3.1. CO_2_ Mitigation Measures in Supply Side

Coal played a major role in supporting the growing electricity demand in China. Nearly all of the emissions growth from power generation has been derived from coal in the recent two decades, although the emissions performance of coal-fired power generation has improved significantly [1]. Based on the generation mix, the measures on CO_2_ mitigation can be divided into three major categories.

#### 3.1.1. Efficiency Improvement of Utilization of Fossil Energy

Efficiency improvement refers to the use of less amount of fossil fuel and CO_2_ emission to produce the same amount of electricity by improving conversion efficiency. This measure is useful for China, where coal is the major energy resource and widely promoted in power sector, thereby, receiving extensive attention at present.

Replacement of backward units with advanced coal-fired generation units with large capacity and high efficiency is an important measure to improve the conversion efficiency. There also exist a number of small-sized, low efficiency coal-fired generation units in China. As a result, this measure still has great potential in more reduction. However, the potential for efficiency improvement and CO_2_ mitigation will continuously decrease as the capacity increases. At this time, more high-efficient generation technologies should be developed like integrated gasification combined cycle and ultra supercritical power generation. In addition, efficiency improvement is expected to reduce CO_2_ emissions for per kilowatt hour (kWh), but the total CO_2_ emissions of power sector in China may still increase continuously as installed capacity has grown quickly in recent years.

#### 3.1.2. Adjust Energy Mix

Replacement of coal with low-carbon fuel or energy with near-zero CO_2_ emission, such as natural gas, renewable energy, and nuclear energy, to adjust energy mix is a significant measure for controlling CO_2_ emissions in the process of power generation.

**(1) Low-Carbon Fuel**

CO_2_ emission of each unit of electricity generated by natural gas is 50%~60% lower than that of traditional thermal power units [22]. Consequently, improvement of utilization ratio of low-carbon fuel like natural gas is a feasible measure of CO_2_ mitigation. However, natural gas resource for generation in China is severely scarce, which contributes little to emission reduction. Thus, the key point to decide whether to put low-carbon fuel into wide use lies in the chance to get stable natural gas supply in low cost or get new gas resource at lower cost.

**(2) Renewable Energy**

Renewable power generation technologies mainly include hydropower, wind power, solar power, biomass power, ocean power, and geothermal power. Generally, most renewable power generation produces CO_2_ during the process of manufacturing equipment and consumables, but no direct CO_2_ emissions arise during power generation process. As a result, it can be seen as near-zero CO_2_ emissions. According to factors regarding technologies and resources, renewable power generation can be developed properly and also serve as an important method of CO_2_ reduction in electricity industry.

China has abundant hydropower resources that remain as the most developed renewable energy resources in the country. The technology of hydroelectricity is relatively mature, of which the installed capacity and generating capacity is the second largest method of power generation next to coal in China. However, large-sized hydropower plants exert some indirect negative impacts on the environment along with the vigorous development of hydropower. Wind energy resource in China is densely located in western, northern, and coastal areas, which is appropriate for centralized development. Similar to wind energy resource, solar energy resource is mainly in western and northern areas. With the development of solar technology, solar energy development is fastened in our country. Low in cost, biomass energy is developed rapidly but with problems in limited resource, collection of biomass, and equipment manufacture. So it should be developed, accordingly. Besides, renewable energy like geothermal energy and ocean energy has certain irreplaceable status in certain area, which leads to fast development in research.

However, the impact on grid stability must be taken into deep consideration, as the connected renewable energy may bring fluctuations into the grid. For instance, wind and sun energy bring along strong fluctuations on a daily and seasonal basis. When the proportion of wind energy is too large, it leads to strong fluctuations in power grid [23].

**(3) Nuclear Power**

Nuclear power, a relatively mature technology, is applied to electricity generation for the remarkable advantages of low operating costs and near-zero CO_2_ emissions. However, it has also encountered barriers, which is mainly related to the public safety like nuclear weapons proliferation and waste management. Chinese technology on nuclear power also requires further development. After over 20 years of development, the basis of China's nuclear industry gradually formed. At present, nuclear power has entered a period of rapid development, and meanwhile the security is always on the primary position.

#### 3.1.3. CCS Technologies

CCS is a process, in which CO_2_ is separated from industrial or energy production chain, then transported to a storage location, and isolated from the atmosphere for a long period of time. It is widely recognized as an exceptional technology in global mitigation, because of its huge potential of an 85% to 90% reduction of CO_2_ emissions in thermal power stations [24]. So far, CCS demonstration projects have been constructed in several thermal power stations of Beijing, Shanghai, and some other places. However, they are restricted to small-scale plants. Due to the high investment cost and large energy consumption of CCS under the current technology level, only small-scale projects can be implemented as future technical reserves of CO_2_ mitigation. Since new CCS technology with low cost and energy consumption is the focus of future research, China should track related technical updates in order to meet the growing demand of CO_2_ mitigation.

### 3.2. CO_2_ Mitigation Measures in Demand Side

DSM, the most effective measures on mitigation in demand side, is a series of electricity management activities aiming at energy conservation and environmental protection, by optimizing the terminal power consumption mode and improving utilization efficiency. Thus, the power demand and CO_2_ emissions in power industry decrease indirectly. 

In China, the DSM has been explored and carried out since the 1990s. During 1991 to 1995, a number of DSM seminars lectured by international experts were organized in China. During 1996 to 2000, several DSM demonstration projects, such as the applications of peak-valley price, energy-saving lamps, were gradually developed, which accumulated experience for DSM. Especially since 2002, DSM has received extensive attention of the whole society due to the tense relationship between power supply and demand. Since then, DSM has entered a period of rapid development in China. New policies on DSM have been released by national and provincial governments, which play an optimistic role in implementing the orderly use of electricity, enhancing energy efficiency, and easing the contradiction between power supply and demand.

Drawing on advanced experience from foreign countries, DSM work can be expanded successfully through the following advices.

1. Governments of all levels should play a leading role in creating a conducive environment for DSM.
2. An effective incentive for stable financial support for carrying out DSM should be quickly set up.
3. Electric power companies should play a dominant role in DSM extension and application.
4. Energy-saving intermediary organizations can help to form a market mechanism of energy conservation.

### 3.3. CO_2_ Mitigation Measures in Power Grid

Power grid is not only a bridge connecting supply side and demand side physically, but also an important medium of achieving mitigation benefits of both sides. The smart grid is considered as a way to reduce energy consumption, improve the electricity network efficiency, and manage renewable energy generation. Besides, it provides accesses for nonfossil energy to get into the grid, including nuclear power, hydropower, wind power and other near-zero emission energy. Briefly speaking, smart grid is an important mean to realize energy conservation and emission reduction, as well as the climate change mitigation.

**Figure 3:**
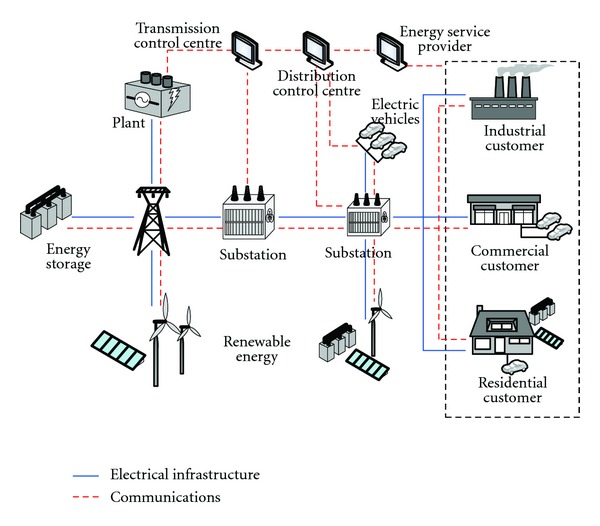
The smart grid chart.

Smart grid is an electricity network that uses digital and other advanced technologies to monitor and manage the electricity transmission from all generation sources to meet the varying electricity demands of end-users, as seen in Figure 3. The significance of the smart grid construction in the promotion of energy conservation and the development of low-carbon economy is as follows.

1. Large-scale clean energy units are allowed to get connected into the grid speed up the development of clean energy promote the optimization, and adjustment of energy mix.
2. Consumers are guided to arrange time duration of electricity consumption, cut down the peak load and the coal consumption in a reasonable manner.
3. Line losses will decrease remarkably in transmission due to the applications of advanced technologies, including UHV, flexible transmission, low-carbon power dispatch, and distributed generation, as well as dual-direction interaction between consumers and the grid.
4. Effective interaction between the grid and consumers will be achieved. The promotion of energy-saving technologies will improve the power consumption efficiency.
5. Large-scale applications of electric vehicles will be promoted. Low-carbon economy will get improved and the mitigation benefits will be achieved.

State Grid Corporation is committed to building a strong smart grid in China, in which UHV power grid is taken as the backbone, and grids at all levels develop coordinately. Plans for a pilot smart grid were outlined in 2010, programming the extension deployment to 2030. Investments in the smart grids will have reached at least USD 96 billion by 2020 [25].

## 4. The Construction of IOCM

**Figure 4:**
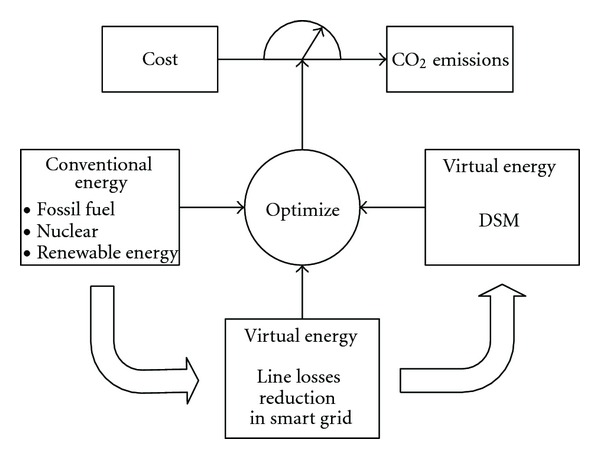
Schematic Diagram of IOCM.

Since any single measure is far from the goal of CO_2_ mitigation in power sector, all feasible options must be taken into consideration. In this section, equivalent virtual energy, consisting of the energy saving capacity in both demand side and the power grid, together with the conventional energy in supply side is unified planning for IOCM. Objective function of IOCM denotes the lowest cost and CO_2_ emissions. Finally, the optimal plan of energy distribution, considering both economic benefits and mitigation benefits, can be figured out by multiobjective optimization calculations (Figure 4). 

Virtual energy in power grid works mainly through smart grid technologies, such as UHV, low-carbon power dispatch, and distributed generation, bringing less line loss. The energy, coming from line losses reduction, is regarded as the smart grid virtual energy (SGVE). Virtual energy in demand side works through DSM, mainly including energy-saving lamps (LVE), energy-saving motors (MVE), energy-saving transformers (TVE), frequency converter (FCVE), and efficient appliances (AVE). For instance, all DSM programs, aiming at promotion of energy-saving lamps, can be gathered up as LVE.

### 4.1. Objective Functions

The lowest-cost objective function denotes the minimum net present value of the total cost, when meeting the electricity demand. It can be expressed by

(1) ⁡min⁡ fCOST=∑y=1Y{∑n=1N[ACIyn(Cyn−Cn0)+COyn·Pyn·8760]  ×1(1+r)y},

where *y* is the *y*th year of studied; *Y* is the total number of years studied; *n* is the *n*th type of energy source; *N* is the total types of energy source, including fossil fuel, nuclear, renewable energy and virtual energy; ACI_*yn*_ is the annual value of the investment cost of the *n*th type of energy per unit capacity in the *y*th year, China Yuan/kilowatt (CNY/kW); CO_*yn*_ is the operating cost of the *n*th type of energy per unit generation in the *y*th year, China Yuan/kilowatt hour (CNY/kWh); *C*_*yn*_ is the installed capacity of the *n*th type of energy in the *y*th year, kilowatt (kW); *C*_*n*_^0^ is the existing installed capacity of the *n*th type of energy, kW; *P*_*yn*_ is the average power generation output of the *n*th type of energy in the *y*th year, kW; *r* is the discount rate.

The lowest-emissions objective function denotes the lowest CO_2_ emissions, when meeting the electricity demand. It can be expressed by

(2) $$\begin{array}{c} \min f_{{\text{CO}}_{2}}=\sum_{y=1}^{Y}\sum_{n=1}^{N}P_{yn}\cdot 8760\cdot \theta_{yn}, \end{array}$$

where *θ*_*yn*_ is the CO_2_ emissions coefficient of the *n*th type of energy in the *y*th year tonnes/kWh. Value of *θ*_*yn*_ decreases with the further efficiency improvement of utilization of fossil energy and wider applications of CCS technologies.

Optimal plans of energy distribution based on (1) and (2) are quite different. The optimal plan based on (1) contains a mass of low-cost energy, while that these based on (2) contains a mass of clear energy instead. However, contradict results in a certain extent, and the fuzzy multiobjective planning method is used here to solve this problem.

### 4.2. Constraint Conditions

**(1) Electricity Demand Constraints**

The electricity generation of both conventional and virtual energy is not less than the predicted electricity demand

(3) $$\begin{array}{c} \sum_{n=1}^{N}P_{yn}\cdot 8760\geq E_{y}\;\left(y=1,2,\ldots ,Y\right), \end{array}$$

where *E*_*y*_ is the predicted value of electricity demand in the *y*th year kWh.

**(2) Peak Load Constraints**

The total installed capacity of both conventional and virtual energy is not less than the sum of peak load and reserve capacity

(4) $$\begin{array}{c} \sum_{n=1}^{N}C_{yn}\geq \left(1+R\right)\cdot D_{y}\;\left(y=1,2,\ldots ,Y\right), \end{array}$$

where *D*_*y*_ is the predicted value of peak load in the *y*th year, kW; *R* is the coefficient of reserve capacity.

**(3) Generation Output Constraint**

The annual electricity generation of each type of energy cannot exceed its upper limit

(5) $$\begin{array}{c} P_{yn}\cdot 8760\leq C_{yn}\cdot T_{n}\;\left(n=1,2,\ldots ,N;\;\;y=1,2,\ldots ,Y\right), \end{array}$$

where, *T*_*n*_ is the annual utilization hours of the *n*th type of energy, hour.

**(4) Installed Capacity Constraints**

Due to the technology, funds, policy, and other limits, the annual installed capacity of each type of energy has its upper limit which cannot be exceeded

(6) $$\begin{array}{c} C_{yn}\leq C_{yn}^{\max}\;\left(n=1,2,\ldots ,N;\;\;y=1,2,\ldots ,Y\right), \end{array}$$

where *C*_*yn*_^max⁡^ is the maximum allowable capacity of the *n*th type of energy in the *y*th year, kW.

**(5) Energy Mix Constraints**

According to the planning requirements, the proportion of nonfossil energy generation in the *y*th year has its lower limit

(7) $$\begin{array}{c} \sum_{n=ma}^{mb}P_{yn}\geq \alpha_{y}\cdot \sum_{n=1}^{mb}P_{yn}\;\left(y=1,2,\ldots ,Y\right), \end{array}$$

where the first type to *mb*th type is conventional energy; the *ma*th type to *mb*th type is nonfossil energy; *α*_*y*_ represents the minimum proportion of nonfossil energy in total electricity generating in the *y*th year.

**(6) CO_2_ Emissions Constraints**

Annual CO_2_ emissions should not exceed the maximum allowable emissions

(8) $$\begin{array}{c} \sum_{n=1}^{N}P_{yn}\cdot 8760\cdot \theta_{yn}\leq M_{y}\;\left(y=1,2,\ldots ,Y\right), \end{array}$$

where *M*_*y*_ is the upper limit of CO_2_ emissions in the *y*th year tonnes.

## 5. Case Study

According to the “12th Five-Year Plan” (TFP), the peak load, the total electricity consumption, and installed capacity are expected to reach 1040 gigawatts (GW), 6270 TWh, and 1437 GW, respectively, in 2015. Moreover, the peak load, the total electricity consumption, and installed capacity are expected to reach 1377 GW, 8200 TWh, and 1885 GW, respectively, in 2020 [20]. The power demand and installed capacity for each type of energy forecasting in 2015 and in 2020 are shown in Table 1.

IOCM is applied to the optimization of energy sources in 2015 and in 2020, respectively. In this model, 7 types of conventional energy sources, including coal, gas, hydro, nuclear, wind, solar, and biomass, and 6 types of virtual energy sources, including LVE, MVE, TVE, FCVE, AVE, and SGVE, are taken into consideration. The main parameters, presented in Table 2 come from the literatures [17–20] directly or are estimated based on literatures indirectly.

According to the results of IOCM, the total installed capacity will reach 1366 GW in 2015, of which the conventional energy is 1266 GW, while virtual energy is 100 GW. Moreover, the total installed capacity will reach 1837 GW in 2020, of which the conventional energy is 1630 GW, while virtual energy is 207 GW. Installed capacity for each type of conventional energy is shown in Table 3. It is obvious that lower-cost clean energy, such as hydro, wind, and nuclear power will have a rapid development. In comparison with TFP, in 2015, the installed capacity of coal-fired plants will decrease by 93 GW, accounting for 9.97% of the total installed capacity of coal-fired plants, while CO_2_ emissions will decrease by 378 million tonnes (Mt), accounting for 10.02% of total emissions. Moreover, in 2020, installed capacity of coal-fired plants will decrease by 145 GW, accounting for 12.50% of the total installed capacity of coal-fired plants, while CO_2_ emissions will decrease by 573 million tonnes (Mt), accounting for 12.59% of the total emissions. The result of IOCM in comparison with TFP is shown in Table 4. As forecasted in Table 5, in 2015, CO_2_ mitigation of virtual energy will reach 228 Mt, accounting for 60.39% of the total CO_2_ mitigation. In 2020, CO_2_ mitigation of virtual energy will reach 421 Mt, accounting for 73.43%.

Consequently, promoting the development of smart grid constructions and DSM program, as well as adjusting the energy mix and improving energy efficiency are the most effective measures on CO_2_ mitigation. The optimization of the CO_2_ mitigation of power sector, under the premise of meeting the demand on electricity, leads to less CO_2_ mitigation and installed capacity. Moreover, this could be a good solution to problems that are caused by factors like resources, capital, environmental, and other things and helps to achieve the goal of sustainable development.

## 6. Conclusions

Power sector is under tremendous pressure of CO_2_ mitigation. Based on the discussion of all feasible measures, IOCM is proposed, which integrates the equivalent virtual energy, consisting of the energy saving capacity in both demand side and the power grid, with conventional energy in supply side. Then, the optimal plan of energy distribution, considering both economic benefits and mitigation benefits, can be figured out by multiobjective optimization calculations. The main conclusions are as follows.

1. Development of measures on CO_2_ mitigation should depend on the mature degree of technology. Large-sized replacement of small-sized coal-fired generation units with high efficient large-sized units is a mature technology deserving wide promotion. In the short and mediumterm, emphasis should be put on mature and low-cost generation technologies, such as hydropower, nuclear power, and wind power. Through more research funds, obstacles in the development of solar and CCS energy could be removed in the medium and long term.
2. Since any single measure is far from the goal of CO_2_ mitigation in power sector, emphasis should be put on the integrated application of various measures, including adjusting energy mix, improving energy efficiency in supply side, as well as the energy saving in demand side and power grid.
3. Based on the data from TFP, IOCM is applied to the optimization of power energy resources in power sector in 2015 and in 2020. CO_2_ mitigation is indirectly achieved by the development of DSM and smart grid constructions which lead to less demand and loss. The results indicate that development of DSM and the smart grid can make great contributions to CO_2_ mitigation of power sector in China, reducing the CO_2_ emissions by 10.02% and 12.59%, respectively, in 2015 and in 2020.

## Figures and Tables

**Table 1:** Power demand and installed capacity for each type of energy in TFP [20].

	Power demand		Installed capacity							
	Electricity consumption (TWh)	Peak load (GW)	Coal (GW)	Gas (GW)	Hydro (GW)	Nuclear (GW)	Wind (GW)	Solar (GW)	Biomass (GW)	Total (GW)
2015	6270	1040	933	30	325	43	100	2	3	1437
2020	8200	1377	1160	40	390	90	180	20	5	1885

**Table 2:** Main parameters in IOCM [17–20].

	Life (years)	Investment cost (CNY/kW)	CO_*yn*_ (CNY/KWh)	*T*_*n*_ (hours)	*θ*_*yn*_ (gram/kWh)	
					2015	2020
Coal	25	3724	0.3	5211	769	746
Gas	25	3222	0.32	2938	384.5	373
Hydro	40	4500	0.12	3424	0	0
Nuclear	50	11074	0.08	7861	0	0
Wind	30	9500	0.08	2047	0	0
Solar	25	15000	0.08	932	0	0
Biomass	25	10000	0.4	4356	0	0
LVE	2	433	0	2500	0	0
MVE	10	1439	0	4000	0	0
TVE	30	15152	0	4000	0	0
FCVE	10	5000	0	2500	0	0
AVE	10	3000	0	2000	0	0
SGVE	1	300	0	8760	0	0

**Table 3:** Installed capacity for each type of conventional energy in IOCM (GW).

	Coal	Gas	Hydro	Nuclear	Wind	Solar	Biomass	Total
2015	840	26	325	43	30	0.3	1.7	1266
2020	1015	30	390	90	100	2	3	1630

**Table 4:** The results of IOCM in comparison with TFP.

	2015			2020		
	Installed capacity of coal-fired plants (GW)	Electricity generation (TWh)	CO_2_ emissions (Mt)	Installed capacity of coal-fired plants (GW)	Electricity generation (TWh)	CO_2_ emissions (Mt)
TFP	933	6713	3773	1160	8770	4553
IOCM	840	5973	3395	1015	7636	3980
Reduction	93	740	378	145	1134	573

**Table 5:** CO_2_ mitigation of virtual energy.

	2015		2020	
	Mitigation (Mt)	Proportion	Mitigation (Mt)	Proportion
LVE	77.15	20.41%	195.75	34.16%
MVE	6.40	1.69%	13.52	2.36%
TVE	83.94	22.21%	81.43	14.21%
FCVE	31.91	8.44%	81.13	14.16%
AVE	19.23	5.09%	36.70	6.41%
SGVE	9.63	2.55%	12.22	2.13%

Total	228.26	60.39%	420.75	73.43%
